# Screw stripping and its prevention in the hexagonal socket of 3.5-mm titanium locking screws

**DOI:** 10.1038/s41598-021-00720-w

**Published:** 2021-10-29

**Authors:** Hyo-Jin Lee, Young Uk Park, Sung Jae Kim, Hyong Nyun Kim

**Affiliations:** 1 Department of Orthopedic Surgery, Seoul St. Mary’s Hospital, College of Medicine, The Catholic University of Korea, Seoul, Republic of Korea; 2 Department of Orthopedic Surgery, Ajou University Hospital, Ajou University School of Medicine, Suwon, Gyeonggi-do Republic of Korea; 3 Department of Orthopedic Surgery, Dongtan Sacred Heart Hospital, Hallym University College of Medicine, Hwaseong, Republic of Korea; 4 Department of Orthopedic Surgery, Kangnam Sacred Heart Hospital, Hallym University College of Medicine, 948-1, Dalim-1dong, Youngdeungpo-gu, Seoul, 150-950 Republic of Korea

**Keywords:** Health care, Medical research

## Abstract

There have been several reports about the difficulties in removing 3.5-mm titanium locking screws from plates due to the stripping or rounding of the hexagonal screw socket. We investigated whether stripping the locking screw sockets can be prevented by using different screwdrivers or interposing materials into the socket during removal. We overtightened 120 3.5-mm titanium locking screws (Depuy Synthes, Paoli, PA) equally into locking plates on sawbone tibia models, applying a uniform torque of 4.5 Nm, exceeding the recommended torque of 1.5 Nm. Twenty screws each were removed using a straight-handle 2.5-mm screwdriver, T-handle screwdriver, hex key wrench, and straight-handle screwdriver with a non-dominant hand. In addition, 20 screws were removed using foil from a suture packet inserted into the screw socket or using parts of a latex glove inserted into the screw socket. The incidence rates of screw stripping using the straight-handle screwdriver, T-handle screwdriver, hex key wrench, non-dominant hand, foil interposition, and latex glove interposition were 75%, 40%, 35%, 90%, 60%, and 70%, respectively. When a T-handle screwdriver or hex key wrench was used, the probability of screw stripping was 4.50 times (odds ratio = 4.50, 95% confidence interval = 1.17 to 17.37, *p* = 0.03) and 5.57 times (odds ratio = 5.57, 95% confidence interval = 1.42 to 21.56, *p* = 0.01) lower than that with the straight-handle screwdriver, respectively. Foil or latex glove interpositions did not prevent screw stripping. Thus, in the current experimental study, T-handle screwdriver or hex key wrench usage decreased the incidence rate of screw stripping during removal compared to straight-handle screwdriver use.

## Introduction

Locking plates can provide high fixation stability for reliable healing in various fractures. These plates are usually manufactured from titanium alloys with higher biocompatibility, higher fatigue strength, and lower risk of infection and are isoelastic with the bone compared to stainless steel^1–7^. However, titanium is prone to cross-threading or cold welding under strong metal-to-metal contact^7–9^. There have been several reports about the difficulties in removing 3.5-mm titanium locking screws from the locking plates after healing of the fractured bone^3,5,10–16^. Removal is challenging when the hexagonal socket of the screw is stripped, rendering the drivers useless. Various techniques have been suggested to remove stripped locking screws^6,9,14,15,17,18^. However, once the screw is stripped, the surgery duration is prolonged, despite the broken screw or stripped screw removal techniques, which increases the possibility of complications such as more bleeding, infection, neurovascular injury, and even iatrogenic fractures^4,9,11^. Therefore, the prevention of screw stripping is essential.

Appropriate use of aiming sleeves and torque-limiting screwdrivers is important to prevent cross-threading or overtightening of the screws during insertion. However, there are few suggestions that can be followed during screw removal to prevent screw stripping^7,19^. Some authors suggest the insertion of aluminum foil or part of a sterile glove into the screw socket to increase the friction between the driver and the socket; nevertheless, these tips are anecdotal, and none have been proven effective^4,19–22^. Currently, star-shaped (hexalobular) sockets, which are less prone to stripping, are now widely used for locking screws^4,23^. However, conventional locking screws with hexagonal sockets, widely used in the past and still used, may require removal someday.

We performed an experimental study on sawbone tibia models to determine whether the incidence of stripping of the hexagonal socket of 3.5-mm titanium locking screws (Depuy Synthes, Paoli, PA) can be minimized by using a T-handle driver with a relatively larger dimension of the driver tip or a hex key wrench. We also tested whether there was a difference in the incidence of screw stripping by using the dominant hand and the non-dominant hand. In addition, we tested whether the interposition of the aluminum foil or parts of the latex glove into the screw socket effectively reduces the incidence of screw stripping.

## Materials and methods

### Preparation of jammed locking screws into the locking plates

**Figure 1:**
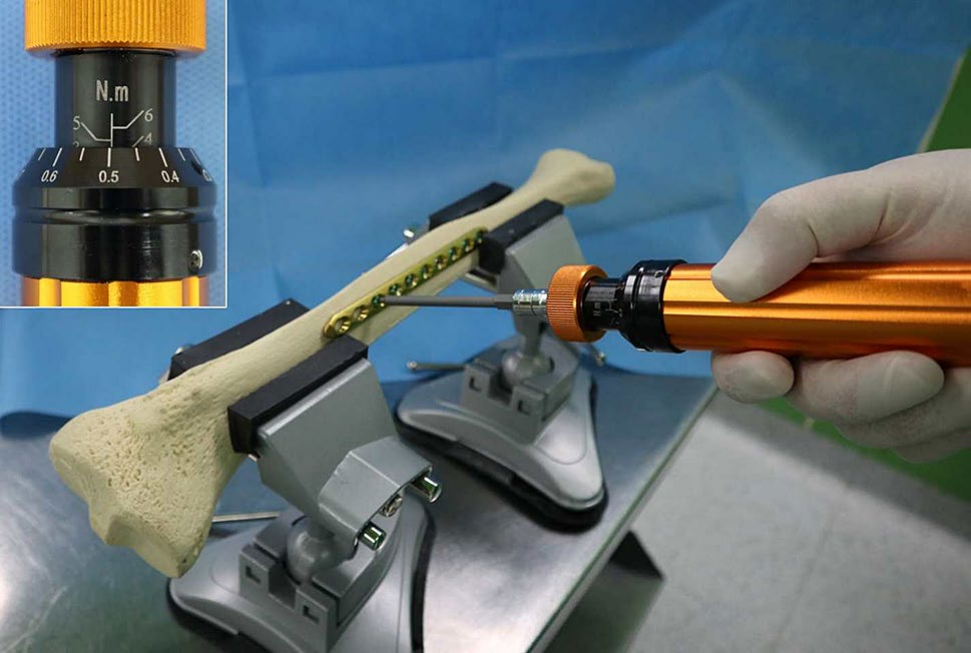
To overtighten and jam the locking screws into the locking plates by applying an identical torque, a torque driver with the torque limit set at 4.5 Nm was used (upper left box). A total of 120 screws were overtightened by applying an identical torque of 4.5 Nm, which exceeded the recommended torque of 1.5 Nm for the 3.5-mm locking screw insertion.

We prepared 6 sawbone tibia models and 12 3.5-locking compression plates (LCPs, Depuy Synthes) with 10 holes each and 120 3.5-mm titanium locking screws (Depuy Synthes). Two plates were attached to each sawbone tibia model. To overtighten and jam the locking screws into the locking plates with the same amount of torque, a torque driver with the torque limit set at 4.5 Nm was used. A total of 120 screws were overtightened by applying a torque of 4.5 Nm, which exceeded the recommended torque of 1.5 Nm for the 3.5-mm locking screw insertion (Fig. 1). The torque of 4.5 Nm was finalized by slowly overtightening the locking screw into the locking plate, starting with a torque of 1.5 Nm with subsequent increments of 0.5 Nm. We used a torque driver that could adjust the torque by 0.05 Nm (Fig. 1). When the torque was set at 4.0 Nm, 1 out of 5 (20%) screws was stripped during removal using a straight-handle 2.5-mm driver (Depuy Synthes), and at 4.5 Nm, 3 out of 5 (60%) screws were stripped. We found that over 5.0 Nm, the screw socket was stripped during the insertion. Thus, we set 4.5 Nm as the torque limit for the study.

### Removal of jammed locking screws

**Figure 2:**
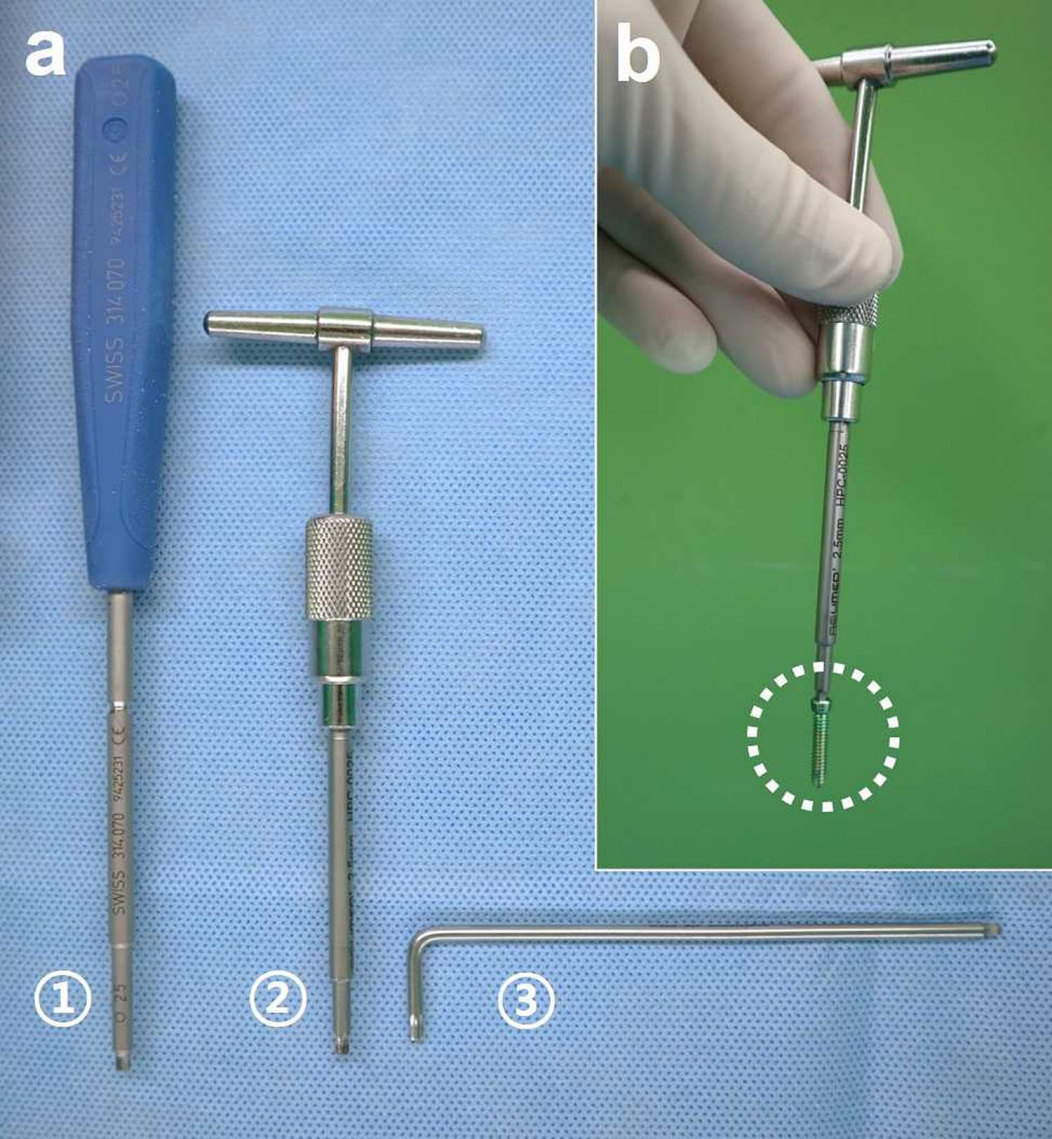
(**a**) Various types of 2.5-mm screwdrivers are presented. A 2.5-mm straight-handle screwdriver (Depuy Synthes, Paoli, PA) (①), a 2.5-mm driver tip (Acumed, Hillsboro, OR) connected to a T-handle (②), and a 2.5-mm hex key wrench (Wera Werkzeuge GmbH, Wuppertal, Germany) (③) were used to remove the locking screws. (**b**) The dimension of the 2.5-mm hexagonal screwdriver tip (Acumed) was slightly larger than that made by the other manufacturer (Depuy Synthes), such that it could hold the 3.5-mm titanium locking screw (Depuy Synthes) without the screw-holding sleeve.

A straight-handle 2.5-mm screwdriver (Depuy Synthes), a T-handle screwdriver (Acumed, Hillsboro, OR), and a hex key wrench (Wera Werkzeuge GmbH, Wuppertal, Germany) were used to remove 20 screws each (Fig. 2).

**Figure 3:**
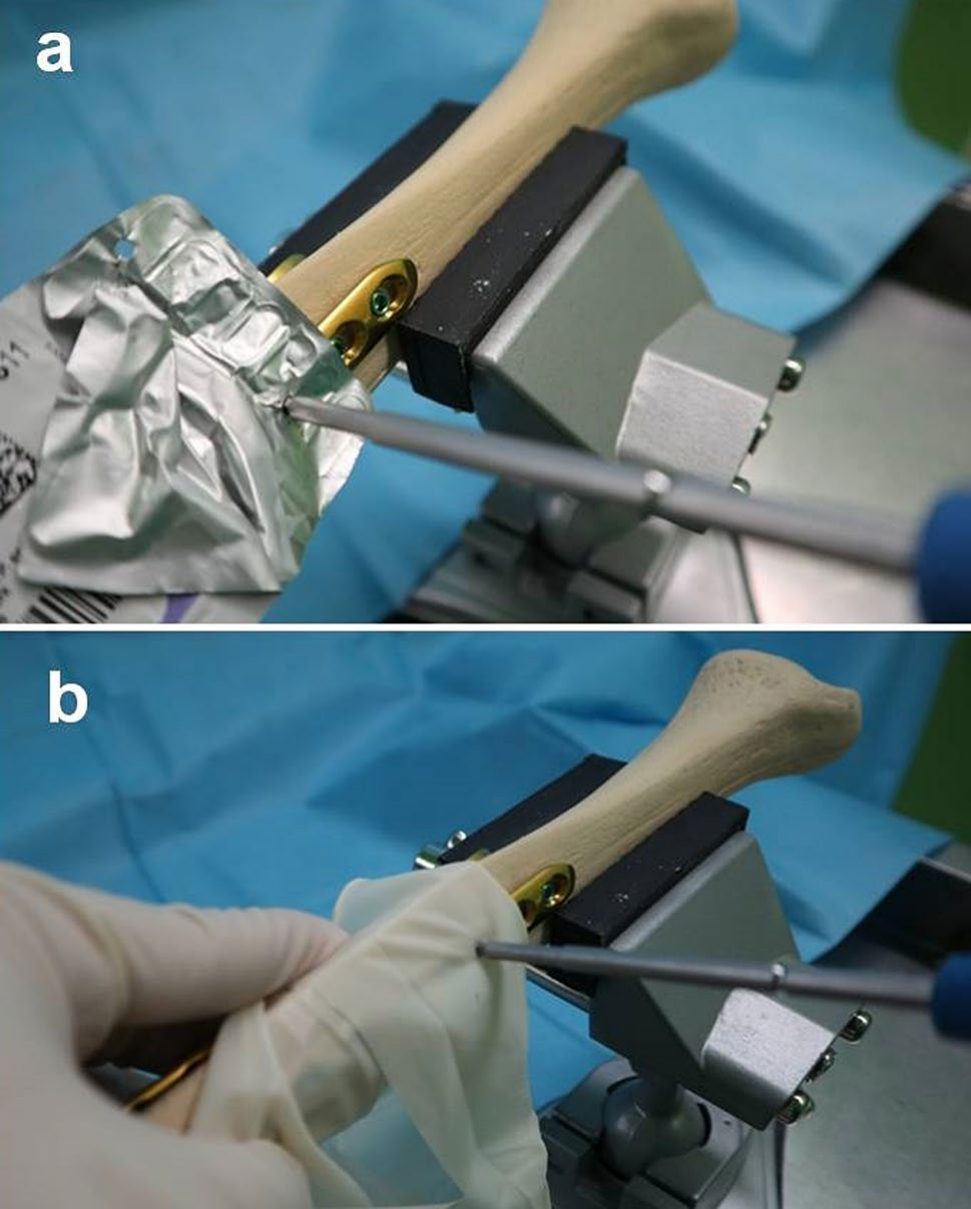
(**a**) Twenty screws were removed with the foil from a suture packet inserted into the screw socket using the straight-handle driver. (**b**) Twenty screws were removed with the parts of a latex glove inserted into the screw socket.

Twenty screws were removed with a straight-handle screwdriver using the non-dominant hand. Twenty screws were removed with a foil from a suture packet inserted into the screw socket, and 20 were removed with parts of a latex glove inserted into the screw socket using the straight-handle driver and the dominant hand (Fig. 3). When the foil was placed over the screw socket and the screwdriver tip was inserted into the socket, the foil was punctured by the driver tip such that the interposition of the foil inside the socket was not possible. Therefore, we wrapped the tip of the driver with the foil to engage the driver tip in the screw socket until it reached full depth.

We counted the number of stripped screws that could not be removed and compared the incidence rate of stripping with different screwdrivers and the interposition of foil or parts of latex gloves. Screw stripping was defined as rounding of the hexagonal screw socket such that the screw could not be removed with the screwdriver.

### Statistical analysis

We performed a pilot study on 20 3.5-mm titanium locking screws jammed into the locking plate to determine the appropriate sample size for the study. As a result, 8 out of 10 (80%) and 3 out of 10 (30%) screws were stripped using the straight-handle driver and the T-handle screwdriver, respectively. Assuming 95% power and a 0.05 significance level, 20 samples in each group were calculated to detect a decrease in the incidence of screw stripping from 80% in the control group to 30% in the experimental group^24^. In a previous study on the correlation between the socket design of the locking screws and screw stripping, 10 screws were used for each group of 9 socket designs^4^. We decided that 20 screws for each group would be appropriate for the study. We used the chi-square test to determine the significance of the association between the binary outcomes (stripped and irremovable screws/removed screws) and the use of different screwdrivers or the interposition of materials. A chi-square test was performed on the two-way contingency table with the row variable set as the use of different screwdrivers or the interposition of materials (use of straight-handle screwdriver/use of other type of screwdrivers or interposition of materials) and the column variable set as the binary outcomes (stripped and irremovable screws/removed screws). The use of straight-handle screwdrivers was used as the reference. We calculated the odds ratio and its 95% confidence interval (95% CI) on this two-way contingency table to measure the association between the use of different screwdrivers or interposition of materials and prevention of screw stripping. We used Fisher's exact test to analyze whether the non-dominant hand was associated with a high incidence of screw stripping. Statistical significance was set at *p* < 0.05. Statistical analysis was performed using SPSS version 22.0 (IBM Corporation, Armonk, NY, USA).

### Ethics approval

This study was approved by the IRB of Hallym University Kangnam Sacred Heart Hospital (IRB-2020-12-015). The study did not involve humans or animals.

## Results

**Figure 4:**
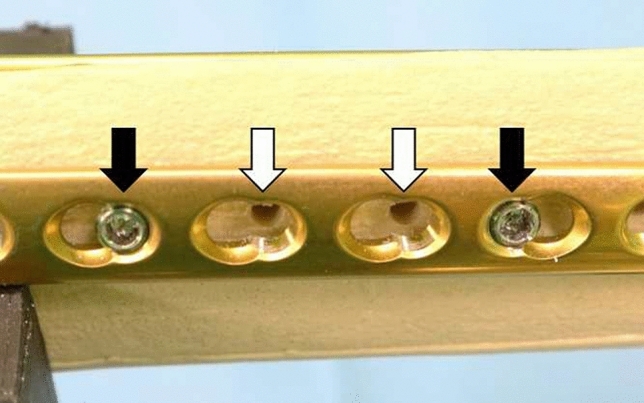
Using a straight-handle screwdriver (Depuy Synthes), 5 (25%) jammed 3.5-mm titanium locking screws (Depuy Synthes) could be removed from the plate (white arrows). However, 15 (75%) screws were stripped and could not be removed (black arrows).

The results are presented in Table 1. When the straight-handle screwdriver, T-handle screwdriver, and hex key wrench were used to remove the 3.5-mm titanium locking screws, 15 (75%), 8 (40%), and 7 (35%) screws were stripped (Fig. 4).

**Table 1:** Incidence of stripping during removal of jammed screws.

	Straight-handle screwdriver	T-handle screwdriver	Hex key wrench	Nondominant hand	Foil interposition	Latex glove interposition
	(n = 20)	(n = 20)	(n = 20)	(n = 20)	(n = 20)	(n = 20)
**Stripped screws**	15 (75%)	8 (40%)	7 (35%)	18 (90%)	12 (60%)	14 (70%)
**Removed screws**	5 (25%)	12 (60%)	13 (65%)	2 (10%)	8 (40%)	6 (30%)

### Values are given as the number and the percentage in parentheses.

The probability of stripping the screw was reduced by 4.50 times (odds ratio = 4.50, 95% CI = 1.17 to 17.37, *p* = 0.03), and 5.57 times (odds ratio = 5.57, 95% CI = 1.42 to 21.56, *p* = 0.01) when the T-handle screwdriver and the hex key wrench were used, respectively, compared to that using the straight-handle screwdriver (Table 2). Foil interposition or latex glove interposition during screw removal did not prevent screw stripping (Table 2).

**Table 2:** Association between the use of different screwdrivers or interposition of materials and prevention of screw stripping.

	Univariate analysis: chi-square and OR		
	OR	95% CI	*p* value
**Use of straight-handle driver**	Reference		
**Use of T-handle driver**	4.50	1.17–17.37	0.03
**Use of hex key wrench**	5.57	1.42–21.86	0.01
**Foil interposition**	2.00	0.52–7.72	0.31
**Latex glove interposition**	1.29	0.32–5.18	0.72

OR = odds ratio, 95% CI = 95% confidence interval.

When the non-dominant hand was used with the straight-handle screwdriver, the incidence rate of screw stripping increased compared to when using the dominant hand. However, the difference was not statistically significant (90% vs. 75%, *p* = 0.41) (Tables 2,3). The probability of stripping the screw was reduced by 13.50 times (odds ratio = 13.50, 95% confidence interval = 2.43 to 74.87, *p* = 0.002), and 16.71 times (odds ratio = 16.71, 95% confidence interval = 2.97 to 93.89, *p* = 0.001) when the dominant hand was used with the T-handle screwdriver and the hex key wrench, respectively, compared to that using the non-dominant hand with the straight-handle screwdriver (Table 3).

**Table 3:** Association between the use of a dominant hand and prevention of screw stripping.

	Univariate analysis: chi-square and OR		
	OR	95% CI	*p* value
**Use of nondominant hand with straight-handle driver**	Reference		
**Use of dominant hand with straight-handle driver**	3.00	0.51–17.74	0.41
**Use of dominant hand with T-handle driver**	13.50	2.43–74.87	0.002
**Use of dominant hand with hex key wrench**	16.71	2.97–93.89	0.001

OR = odds ratio, 95% CI = 95% confidence interval.

## Discussion

**Figure 5:**
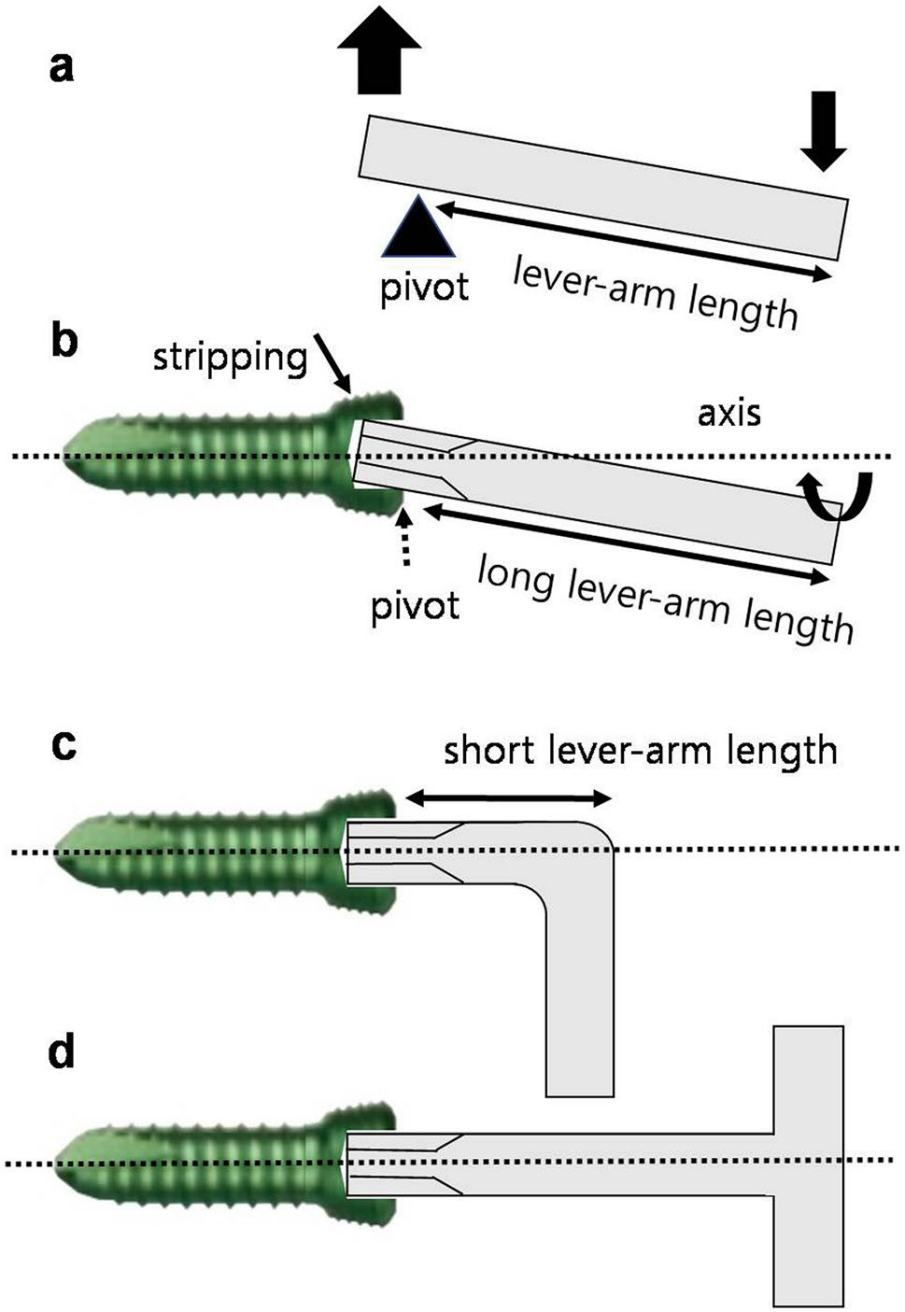
(**a**) With a long lever arm, a greater force can be produced with a smaller force across the pivot. (**b**) When a high torque during counterclockwise rotation force is required to remove a jammed locking screw, a straight handle is relatively thin for a full grip compared to the T-handle, and the axis of the straight-handle driver may not be aligned with the axis of the screw. This may cause the driver tip to deviate inside the screw socket. When the driver shaft is long, it can work as a long lever arm to create a greater force at the driver tip to ream and strip the socket. (**c, d**) The hex key wrench and T-handle driver can transfer the counterclockwise rotation force to the screw with less deviation of the axis of the driver tip from the axis of the screw, which can maximize the contact area between the driver tip and the screw socket to prevent screw stripping.

The LCP (Depuy Synthes) has been introduced as a new standard of AO plating osteosynthesis. It is widely used for various fractures, such as comminuted tibia fractures, or even in joint sparing salvage surgery after articular oncological resections^3–7,25^. However, there are reports of difficulties in removing 3.5-mm titanium locking screws due to stripping of hexagonal sockets^3,5,10,16^. This may be because titanium is more malleable than stainless steel, and the difference in the mechanical resistance between the stainless-steel screwdriver and the titanium screw may lead to stripping of the titanium screw. Titanium has a high degree of biocompatibility, which leads to increased osteointegration and thus a higher risk of screws becoming encased^17,26^. Furthermore, LCP is used for the minimally invasive plate osteosynthesis technique. Hence, when the locking screw hole of the plate is not well exposed, the locking screw may be inserted at the wrong angle and jam into the plate^22^. During screw removal, the screwdriver may sometimes engage with the screw at an incorrect angle, or the driver tip may not be fully engaged in the screw socket before the screwdriver is turned. The surgeon can feel the first slippage of the screwdriver inside the socket. A biomechanical study demonstrated up to a 50% decrease in the maximal torque values after a single event of screwdriver slippage^13^. Although the straight handle is easy to rotate when there is no considerable resistance, as in screw insertion, when a high torque for the counterclockwise rotation force is required to remove a jammed locking screw, the straight-handle screwdriver is relatively thin for a full grip compared to the T-handle. The axis of the straight-handle driver may not be in alignment with the axis of the screw when considerable wrist movement is required in addition to finger movement to rotate the screwdriver. This may cause the tip of the driver to deviate inside the screw socket (Fig. 5). When the driver tip is not evenly in contact with the socket because of this deviation and is in contact only with certain parts, the stress is concentrated at the point of contact. The driver tip reams that part of the socket leading to stripping. Compared to the straight-handle screwdriver, the T-handle screwdriver and hex key wrench can transfer the counterclockwise rotation force to the screw with less deviation of the axis of the driver tip from the axis of the screw. This can maximize the contact area between the driver tip and the screw socket to prevent screw stripping.

However, for convenience during screw insertion, most manufacturers provide a straight-handle driver; moreover, the 2.5-mm straight-handle screwdriver (Depuy Synthes) is commonly provided by the scrub nurse to remove 3.5-mm titanium locking screws (Depuy Synthes). The diameter, length, and design of the 2.5-mm hexagonal driver tip can differ between the products by different manufacturers. Many manufacturers provide a screwdriver shaft that can be connected to the power tool for power insertion of the screws. This screwdriver shaft can be connected to the T-handle. We recommend that surgeons source the largest available 2.5-mm driver tip with sharp edges by testing the engagement of the driver tip in the screw socket, checking how firmly it can hold the screw, and reserving it for screw removal. In some situations, surgeons may have to change their position to use their dominant hand for screw removal. But some may prefer to use their non-dominant hand rather than change their position to use the dominant hand. Because one inadvertent missed turn of the screwdriver with the non-dominant hand can ream the socket, leading to screw stripping, we recommend that surgeons use their dominant hand for screw removal on all occasions^13^.

**Figure 6:**
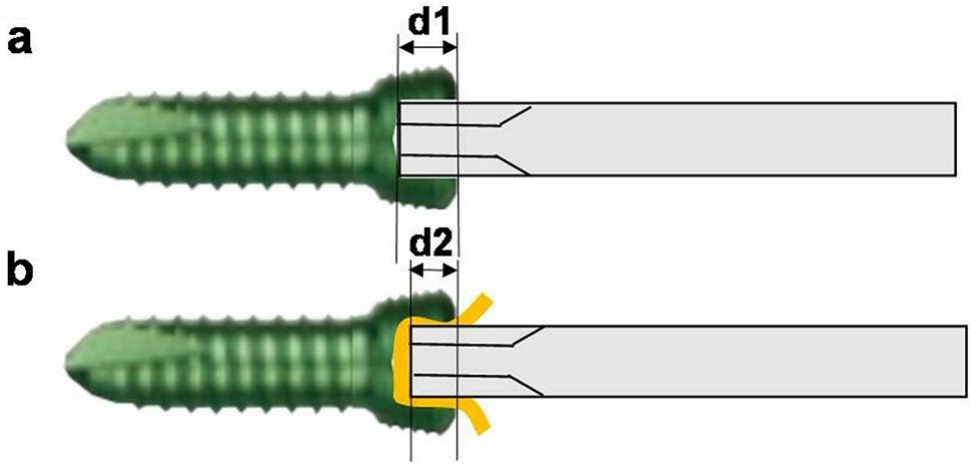
(**a**) The driver tip is engaged with the screw to its full depth (d1). (**b**) When the material is inserted into the screw socket, the driver tip may not be engaged until its full depth (d2 < d1), which can lead to a decrease in the contact area.

This study has some limitations. First, the clinical setting in real patients is different from that in the sawbone model trial. Therefore, we could only simulate the overtightening of the locking screw head into the plate. Osteointegration of the screw threads with the bone could not be simulated^17^. Strong cortical bone ingrowth at the terminal flutes of the screw can cause screw stripping; however, this situation could not be simulated with sawbone models^27^. Second, the amount of torque that leads to screw stripping can be different in different cases. However, for the experimental study, we overtightened all locking screws by applying a 4.5-Nm torque. The results may be different, with different torques applied to overtighten the screw. Third, although the interposition of the foil or parts of the latex glove did not significantly decrease the incidence of screw stripping in the current study, it may potentially be effective with different torques applied on the screws. However, we believe that the interposition of the material can impede the engagement of the driver tip into the screw socket to its full depth, which can lead to a decrease in the contact area (Fig. 6).

Fourth, we tested only the 3.5-mm titanium locking screws, which are the most widely used screws, from one manufacturer. These results may not apply to other screws made by different manufacturers. Moreover, we used new screwdrivers for the current experiment. In the clinical setting, used screwdrivers may have blunt or rounded edges of the hexagonal driver tip resulting in different outcomes. Finally, we acknowledge that without the clinical application and the outcomes, it cannot be concluded that the T-handle driver with a relatively larger 2.5-mm driver tip or the hex key wrench will decrease the incidence of screw stripping. We are now prospectively collecting the clinical results of using the T-handle driver (Acumed) to remove3.5-mm titanium locking screws (Depuy Synthes). There are several screw removal devices that are designed to remove stripped screws^5,15,22^. We suggest that manufacturers should provide a screwdriver specialized for screw removal to prevent screw stripping. This should be equipped with a T-handle and a larger dimension of driver tip, such as 2.6 mm, that can be in complete contact and engagement with the screw socket.

## Conclusion

There have been several reports about the difficulties in removing 3.5-mm titanium locking screws from the locking plate due to the stripping of the hexagonal screw socket. However, there is a lack of research on methods to prevent screw stripping during screw removal. We hypothesized that screw stripping can be prevented by using different screwdrivers or interposing materials into the socket during removal. We performed an experimental study on swabone tibia models, and tested the incidence rate of screw stripping using a straight-handle screwdriver, T-handle screwdriver, hex key wrench, non-dominant hand, foil interposition, and latex glove interposition. In the current experimental study;

- The use of a T-handle screwdriver reduced the incidence of screw stripping by 4.50 times compared to the use of a straight-handle screwdriver.
- The use of a hex key wrench decreased the incidence of screw stripping by 5.57 times compared to the use of a straight handle screwdriver;
- The interposition of foil or parts of the latex glove into the screw socket did not decrease the incidence of screw stripping.

A future study on the surface morphology of stripped screws using emission scanning electron microscopy may enable a more accurate analysis of the effects of different screwdrivers on screw stripping. Furthermore, clinical application results are required to confirm the above findings.

## Data Availability

The datasets used and analyzed during the current study are presented in Table 1.
